# City Mortality

**Published:** 1867-02

**Authors:** 


MORTALITY FOR THE MONTH OF JANUARY.—The subjoined is the mortality report of Health Officer Bridges for the past month:—

CAUSES OF DEATH.

| | | | |
|---|---|---|---|
| Accidents, | 3 | Fever, Remittent, | 3 |
| Bronchitis, | 1 | Fever Purperal, | 1 |
| Burned, | 1 | Fever, Scarlet, | 21 |
| Cancer, | 2 | Fever, Spotted, | 1 |
| Childbed, | 8 | Fever, Typhoid, | 11 |
| Colic, | 1 | Fever, Congestive, | 1 |
| Congestion of Brain, | 3 | Hydrocephelus, | 3 |
| Congestion of Lungs, | 2 | Inflammation of Brain, | 3 |
| Consumption, | 40 | Inflammation of Lungs, | 10 |
| Convulsions, | 38 | Inflammation of Bowels, | 3 |
| Croup, | 11 | Inflammation not stated, | 3 |
| Cold, | 1 | Killed, | 1 |
| Diabetes, | 1 | Old Age, | 11 |
| Disease of Brain, | 1 | Pneumonia, | 1 |
| Disease of Bowels, | 1 | Poisoning, | 1 |
| Disease of Heart, | 6 | Phthisis Pulmonalis, | 2 |
| Disease of Kidneys, | 4 | Rheumatism, | 1 |
| Disease of Liver, | 2 | Stillborn, | 22 |
| Disease of Lungs, | 4 | Scald, | 1 |
| Disease of Spine, | 2 | Small-Pox, | 1 |
| Diarrhœa, | 4 | Suicide, | 2 |
| Diphtheria, | 8 | Teething, | 12 |
| Delirium Tremens, | 2 | Whooping-Cough, | 10 |
| Dropsy, | 5 | Unknown, | 20 |
| Erysipelas, | 2 | | |
| Epilepsy, | 1 | Total, | 299 |

AGES OF THE DECEASED.—Under 5 years, 157; over 5 and under 10 years, 15; over 10 and under 20, 11; over 20 and under 30, 28; over 30 and under 40, 32; over 40 and under 50, 22; over 50 and under 60, 6; over 60 and under 70, 12; over 70 and under 80, 6; over 80 and under 91, 1; unknown, 9. Total, 299.

NATIVITIES.

| | | | | | |
|---|---|---|---|---|---|
| Chicago, | 156 | Germany, | 44 | On the Sea, | 1 |
| Other States, | 39 | Ireland, | 28 | Unknown, | 5 |
| Canada, | 5 | Norway, | 3 | | |
| Denmark, | 1 | Poland, | 1 | Total, | 229 |
| England, | 12 | Scotland, | 4 | | |

DIVISIONS OF THE CITY.

North,......... 93 | South,......... 87 | West,.........119 | Total,......... 299
Total number during the month of December, 309
Total number last year for the month of January, 292

---

PROPOSED NEW HOSPITALS UPON RANDALL'S ISLAND, NEW YORK.—At a semi-monthly meeting, in December last, of the Commissioners of Public Charities and Correction, it was decided to establish a hospital for incurables at the alms-house on Randall's Island. The architect of the Board also received orders to prepare a design for an insane hospital capable of accommodating eighty persons.

